# Comment on “Nationwide trends and disparities in deaths from second- or third-degree atrioventricular block (1999–2022): A 24-year retrospective analysis of CDC WONDER data”

**DOI:** 10.1016/j.ahjo.2026.100981

**Published:** 2026-10-21

**Authors:** S. Sivasankari, S. Senthamarai, J. Han Feliciana, S. Devi

**Affiliations:** a Department of Microbiology, Meenakshi Medical College Hospital & Research Institute, Meenakshi Academy of Higher Education and Research, Enathur, Kanchipuram, Tamil Nadu, 631552, India; b Arulmigu Meenakshi College of Nursing, Meenakshi Academy of Higher Education and Research, India

**Keywords:** Atrioventricular block, Heart block, Mortality trends, CDC WONDER, Death certificate, Cardiovascular mortality, Health disparities, Temporal trends, Geographic disparities, Epidemiology

Dear Editor,

Saeed et al. [1], isolate high-degree atrioventricular block from conduction disorders generally, which is a reasonable thing to do given its distinct clinical course, and they are frank about what death-certificate data cannot show.

The central difficulty is that the authors offer an explanation for their headline finding and then set it aside. Discussing the sharp rise after 2013, they suggest it may partly reflect greater detection and reporting, which “provides more accurate diagnoses, and thus higher mortality rates being reported by physicians on death certificates.” That is a statement that the trend may be an artefact of ascertainment rather than a change in who dies. Having said it, the paper treats the rise as epidemiological throughout, concluding that mortality has increased and that disparities require intervention. The outcome here counts a death whenever a specific conduction code appears anywhere on the certificate as underlying or contributing cause. Any shift in how fully contributing causes are recorded moves the count without altering mortality. The pattern supports this reading: rates rise in every sex, age band, racial group, census region and urbanisation stratum within a few years of one another, which is unusual for a real change in disease burden but expected of a change in coding practice [2]. The authors could test it directly by reporting the mean number of causes listed per certificate by year, or by repeating the analysis restricted to underlying cause. Until that is done, the paper's own alternative explanation remains live, and the abstract and conclusion should say so.

The state ranking compounds the problem. Vermont heads the list at 17.6 per million and Louisiana sits at the bottom at 6.0, a threefold spread [3]. The states identified as highest — Alaska, Idaho, Maine, Rhode Island, Vermont, Washington — are small-population jurisdictions, and the lowest include Florida and New York. Interstate differences in certification practice are well documented and would produce exactly this distribution. Reading it as a disparity in access to pacing, as the discussion does, requires ruling that out first.

Two further points. The non-metropolitan interval for the 1999–2005 decline is given as −10.77 to 4.49, which spans zero and therefore cannot carry *p* = 0.001; one bound is mistyped [4]. And the overall annual percent change for 2013–2022 appears as 6.13 to 9.96 in the abstract and 6.12 to 9.96 in the results. Both intervals are also asymmetric about the stated estimate of 7.26, which warrants a check.

Finally, the pandemic comparison contrasts a three-year mean of 14.9 against a twenty-one-year mean of 9.4. Since rates were climbing steeply from 2013, the earlier average is pulled down by two decades of lower values, so the contrast largely restates the trend. The authors note that no inflection was detected at 2020; that finding is the more informative one.

## CRediT authorship contribution statement

**S. Sivasankari:** Writing – review & editing, Writing – original draft, Visualization. **S. Senthamarai:** Conceptualization, Data curation, Formal analysis. **J. Han Feliciana:** Writing – review & editing, Writing – original draft, Visualization. **S. Devi:** Writing – review & editing, Writing – original draft, Visualization.

## Informed consent

Not applicable.

## Ethical approval

Not applicable (Commentary on a previously published article).

## Declaration of Generative AI and AI-assisted technologies in the writing process

Generative AI tools were used only for language refinement and formatting assistance. All scientific interpretation, critical appraisal, and manuscript preparation were performed independently by the authors.

## Funding

None.

## Declaration of competing interest

The authors declare that there are no conflicts of interest.

## Data Availability

No new data were generated or analyzed in this study.
